# Safety and Immunogenicity of 3 Formulations of an Investigational Respiratory Syncytial Virus Vaccine in Nonpregnant Women: Results From 2 Phase 2 Trials

**DOI:** 10.1093/infdis/jiy065

**Published:** 2018-02-01

**Authors:** Jiři Beran, Jason D Lickliter, Tino F Schwarz, Casey Johnson, Laurence Chu, Joseph B Domachowske, Pierre Van Damme, Kanchanamala Withanage, Laurence A Fissette, Marie-Pierre David, Koen Maleux, Alexander C Schmidt, Marta Picciolato, Ilse Dieussaert

**Affiliations:** 1 Vaccination and Travel Medicine Centre, Hradec Králové, Czech Republic; 2 Nucleus Network, Melbourne, Australia; 3 Klinikum Würzburg Mitte, Standort Juliusspital, Würzburg, Germany; 4 Johnson County Clinic Trials, Lenexa, Kansas; 5 Benchmark Research, Austin, Texas; 6 Department of Pediatrics, SUNY Upstate Medical Center, Syracuse, New York; 7 Clinical Research and Development, GSK, Rockville, Maryland, USA; 8 Research and Development, GSK, Rockville, Maryland, USA; 9 Vaccine and Infectious Disease Institute, University of Antwerp, Antwerp; 10 Biostatistics, Biostat, and Stat Programming; 11 CLS/CIAM; 12 Clinical Research and Development, GSK, Rixensart, Belgium

**Keywords:** Respiratory syncytial virus, RSV, vaccine, women, maternal antibodies

## Abstract

**Background:**

Respiratory syncytial virus (RSV) causes bronchiolitis and pneumonia in neonates and infants. RSV vaccination during pregnancy could boost preexisting neutralizing antibody titers, providing passive protection to newborns.

**Methods:**

Two observer-blinded, controlled studies (RSV F-020 [clinical trials registration NCT02360475] and RSV F-024 [NCT02753413]) evaluated immunogenicity and safety of an investigational RSV vaccine in healthy, nonpregnant 18–45-year-old women. Both studies used a licensed adult formulation of combined tetanus toxoid-diphtheria toxoid-acellular pertussis (Tdap) vaccine as a control. RSV F-020 evaluated immunogenicity and safety: participants were randomized (1:1:1:1) to receive 1 dose of RSV–prefusion F protein (PreF) vaccine containing 30 µg or 60 µg of nonadjuvanted RSV-PreF, 60 µg of aluminum-adjuvanted RSV-PreF, or Tdap. RSV F-024 evaluated safety: participants were randomized 1:1 to receive 1 dose of 60 µg of nonadjuvanted RSV-PreF or Tdap.

**Results:**

Both studies showed similar reactogenicity profiles for RSV-PreF and Tdap. No serious adverse events were considered vaccine related. In RSV F-020, geometric mean ratios of RSV-A neutralizing antibody levels at day 30 versus prevaccination were 3.1–3.9 in RSV-PreF recipients and 0.9 in controls. Palivizumab-competing antibody concentrations increased >14-fold in RSV-PreF recipients on day 30. RSV antibody titers waned after day 30 but remained well above baseline through day 90.

**Conclusions:**

All formulations of RSV-PreF boosted preexisting immune responses in 18–45-year old women with comparable immunogenicity. The RSV-PreF safety profile was similar to that of Tdap vaccine.

## Teaser

A single dose of either 30 µg or 60 µg of nonadjuvanted respiratory syncytial virus prefusion F protein antigen boosts humoral immune responses in women of childbearing age. The investigational vaccine was well tolerated and had a safety profile similar to that of the widely used combined Tdap vaccine.

Respiratory syncytial virus (RSV) is a highly contagious human pathogen that causes respiratory tract infections in all age groups. Protection after infection is short lived, and reinfection is common throughout life [1]. Between 50% and 70% of infants are infected with RSV during their first year of life, and almost all children have experienced RSV infection by their second birthday [1]. RSV disease generally starts as an upper respiratory tract illness and then progresses to lower respiratory tract illness (LRTI), with cough, tachypnea, and wheezing. Bronchiolitis is the signature disease of RSV infection, and some LRTIs progress to pneumonia. Patients may require supplemental oxygen and/or initiation of ventilator support. In high-income countries, RSV infection is the leading cause of hospitalization in infants, with annual rates of 3600–4890 cases per 100000 infants aged <3 months and slightly lower rates in infants aged 3–6 months [2–4]. The burden of RSV disease in ambulatory settings has received less attention, but a study using data from the United Kingdom estimated that rates of medically attended RSV disease among infants <6 months of age were 8.4-fold higher than those for influenza, with a reported 14441 annual RSV-associated episodes per 100 000 infants who visited a general practitioner [4].

Placental antibody transfer can offer protection against RSV, as high titers of maternally derived RSV-neutralizing antibodies are inversely associated with RSV LRTI during the first 6 months of life [5]. Maternal immunization boosts maternal RSV antibody titers and thus has the potential to protect young infants against RSV during the first months of life. Maternal vaccination programs have already proven safe and highly successful in the prevention of neonatal tetanus, pertussis, and influenza [6–8].

The RSV fusion glycoprotein (RSV-F) is a highly conserved surface protein that is essential for virus entry into target respiratory epithelial cells and is implicated in RSV pathogenesis [9]. The F protein is the main target of neutralizing antibody responses and also the target of palivizumab, a licensed monoclonal antibody that is used for passive protection of infants at high risk of severe RSV disease [9, 10]. RSV-F undergoes conformational changes during the fusion of virus envelope and cell membrane, and the prefusion conformation (PreF) exhibits more epitopes for neutralizing antibodies than the postfusion conformation [11]. The RSV-PreF evaluated here is a purified recombinant F protein prepared in Chinese hamster ovary cells and engineered to preferentially maintain the prefusion conformation [12, 13]. In a phase 1 study in healthy adult men [13], RSV-A neutralizing antibody titers increased by 3.2–4.9-fold in recipients of a single 30-μg dose of RSV-PreF adjuvanted to aluminum or a single 60-μg dose of RSV-PreF with or without aluminum adjuvant. Injection site pain, fatigue, and headache were the most frequently reported solicited symptoms and were transient and usually mild to moderate in intensity. Fever was infrequently reported, and no safety concerns were identified that would preclude continued development.

We report the results of 2 phase 2 trials: RSV F-020 (clinical trials registration NCT02360475) and RSV F-024 (NCT02753413). RSV F-020 was a randomized controlled trial that assessed the reactogenicity and immunogenicity of 3 different RSV-PreF formulations in nonpregnant women of childbearing age. Based on day 30 immunogenicity and reactogenicity data from this study, the 60-μg nonadjuvanted RSV-PreF formulation was selected for an additional phase 2 trial (RSV F-024) designed to further evaluate its safety, including hematologic and biochemical parameters, in nonpregnant women of childbearing age.

## METHODS

### Design

RSV F-020 was conducted at multiple sites in Australia, the United States, the Czech Republic, and Germany. RSV F-024 was conducted at a single center in Belgium. Both studies were observer blinded, randomized, and undertaken in accordance with good clinical practice guidelines and the Declaration of Helsinki. Written informed consent was obtained from each subject prior to the performance of any study-specific procedures. A GSK Internal Safety Review Committee monitored RSV F-024 and reviewed unblinded safety data up to 7 days after vaccination.

**Figure 1.**
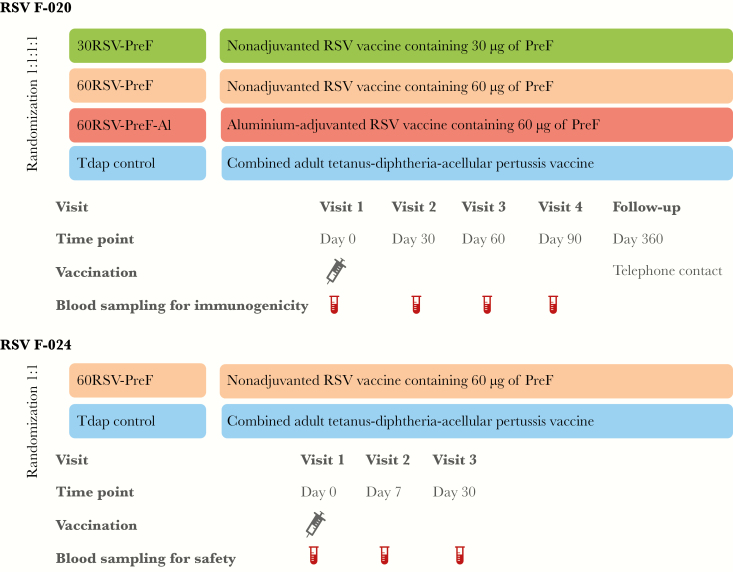
Study design and procedures. PreF, prefusion protein; RSV, respiratory syncytial virus.

Participants in each study were randomized to study groups of equal size at the investigator sites, using a web-based program (Figure 1). The randomization algorithm used a minimization procedure that addressed the age of the participant (18–32 years or 33–45 years). Participants in RSV F-20 received 1 dose of study vaccine containing either 30 µg or 60 µg of nonadjuvanted RSV-PreF (the 30RSV-PreF and 60RSV-PreF groups, respectively), 60 µg of aluminum-adjuvanted RSV-PreF (the 60RSV-PreF-Al group), or an adult formulation of combined tetanus toxoid-diphtheria toxoid-acellular pertussis vaccine (Tdap). Participants in RSV F-024 received 1 dose of 60 µg of nonadjuvanted RSV-PreF (60RSV-PreF-Al group) or Tdap.

Most study sites were located in the northern hemisphere. One site was in Australia. The RSV F-020 trial started in March 2015, and the RSV F-024 trial in April 2016 (ie, following peak seasonal RSV transmission in the northern hemisphere). There were 125 participants enrolled around the beginning of the RSV transmission season in Australia (RSV F-020). Both studies were completed in June 2016.

### Participants

Eligible participants were healthy women 18–45 years of age. Women of childbearing potential were required to use adequate contraception from 30 days before vaccination through 30 days after vaccination in RSV F-024 and through 90 days after vaccination in RSV F-020 and to have a negative pregnancy test on the day of vaccination. Exclusion criteria are provided in the Supplementary Materials.

### Vaccines

The 2 nonadjuvanted (30-μg and 60-µg) vaccine formulations were presented in single-dose vials as lyophilized antigen and were reconstituted using 150 mM sodium chloride solution. In RSV F-020, the 60-μg RSV-PreF-Al vaccine contained 500 μg of aluminum hydroxide as adjuvant and was presented as liquid in 0.5-mL single-dose vials.

The adult Tdap vaccine with aluminum adjuvant (300 µg in the United States and 500 µg in the rest of the world; Boostrix) was used as the control vaccine for both studies. Tdap is currently recommended during the third trimester of pregnancy in 31 countries. All vaccines were manufactured by GSK and were administered intramuscularly into the deltoid region of the upper arm.

### Safety Assessment

Participants recorded solicited injection site and general symptoms on diary cards for 7 days after vaccination. Symptom intensity was graded between 0 and 3, where 0 denoted no symptoms, grade 1 denoted mild symptoms, grade 2 denoted moderate symptoms, and grade 3 denoted severe symptoms. Grade 2 was defined as pain when the arm was moved that interfered with normal everyday activity, redness and swelling (diameter, >50 to ≤100 mm), fever (temperature, >38.5°C to ≤39.5°C), and other symptoms that interfered with normal activity. Grade 3 was defined as significant pain at rest that prevented normal everyday activities, redness and swelling (diameter, >100 mm), fever (temperature, >39.5°C), and other symptoms that prevented with normal activity. All other (unsolicited) adverse events (AEs) were recorded for 30 days after vaccination. Serious adverse events (SAEs) and pregnancies were recorded for the duration of each study. Participants in RSV F-020 were contacted by telephone 1 year after vaccination to determine whether SAEs and pregnancies had occurred since the last study visit.

Hematological and biochemical parameters were measured at days 0, 7, and 30 in RSV F-024 (Figure 1). Toxicity grading from 1–4 was based on Food and Drug Administration (FDA) Guidance [14].

### Immunogenicity Assessment

Immunogenicity was only assessed in RSV F-020; assessment ended 90 days after vaccination (Figure 1).

In brief, RSV-A neutralizing antibodies on Vero cells were quantified using the RSV plaque-reduction neutralization assay. RSV-infected cells were detected using a primary antibody directed against RSV (anti-RSV immunoglobulin G [IgG]) and a secondary antibody conjugated with fluorescein isothiocyanate, allowing the visualization of plaques by immunofluorescence. The serum neutralizing antibody titer was expressed as the estimated dilution 60 (ED60), which corresponds to the inverse of the interpolated serum dilution that yields a 60% reduction in the number of plaques as compared to the virus control wells. The assay cutoff was set at 8 ED60.

An indirect enzyme-linked immunosorbent assay (ELISA) format was used to quantify PreF total IgG and IgG subclass 1 (IgG1) antibodies. A colored product proportional to the amount of anti-PreF protein IgG/IgG1 antibodies present in the test serum was quantified by reading the optical densities (ODs) at 450–620 nm, using a spectrophotometer. The assay cutoffs were set at 10 ELU/mL for PreF IgG and 46.1 ELU/mL for IgG1.

Antibodies specific to site II were quantified by competition ELISA. Palivizumab-like antibodies present in serum samples compete with biotinylated palivizumab for binding to the same epitope on the PreF coated antigen. The OD recorded is inversely proportional to the concentration of the palivizumab-competing antibodies (PCAs) present in the sample. The assay cutoff was set at 3.34 µg/mL.

### Statistical Analysis

The safety analysis was performed on the total vaccinated cohort in each study. Exploratory comparisons of the percentages of participants in RSV F-020 who had any grade 2/3 AE, fever (temperature, >38.5°C), and/or any vaccine-related SAE during the 7-day follow-up period were performed using standardized asymptotic 95% confidence intervals (CIs) to evaluate the difference between groups. In RSV F-024, the percentage of participants with hematologic and biochemical parameters outside of the normal range determined by the local laboratory were tabulated by time point and severity grading and compared to baseline values [14].

The analysis of vaccine immunogenicity in RSV F-020 was performed on the according-to-protocol cohort (all vaccinated subjects meeting eligibility criteria and protocol-defined procedures). Exploratory evaluations compared RSV-PreF vaccine groups in terms of neutralizing anti-RSV-A geometric mean titers (GMTs) and PCA geometric mean concentrations (GMCs) at day 30, using an analysis of covariance model with prevaccination titer and vaccine group as covariates. Pairwise comparisons were made using the Tukey multiple comparison adjustment. Participants with antibody titers and concentrations below the cutoff of the respective assays were given an arbitrary value of half the cutoff, for the purposes of calculation GMTs and GMCs, respectively.

## RESULTS

**Figure 2.**
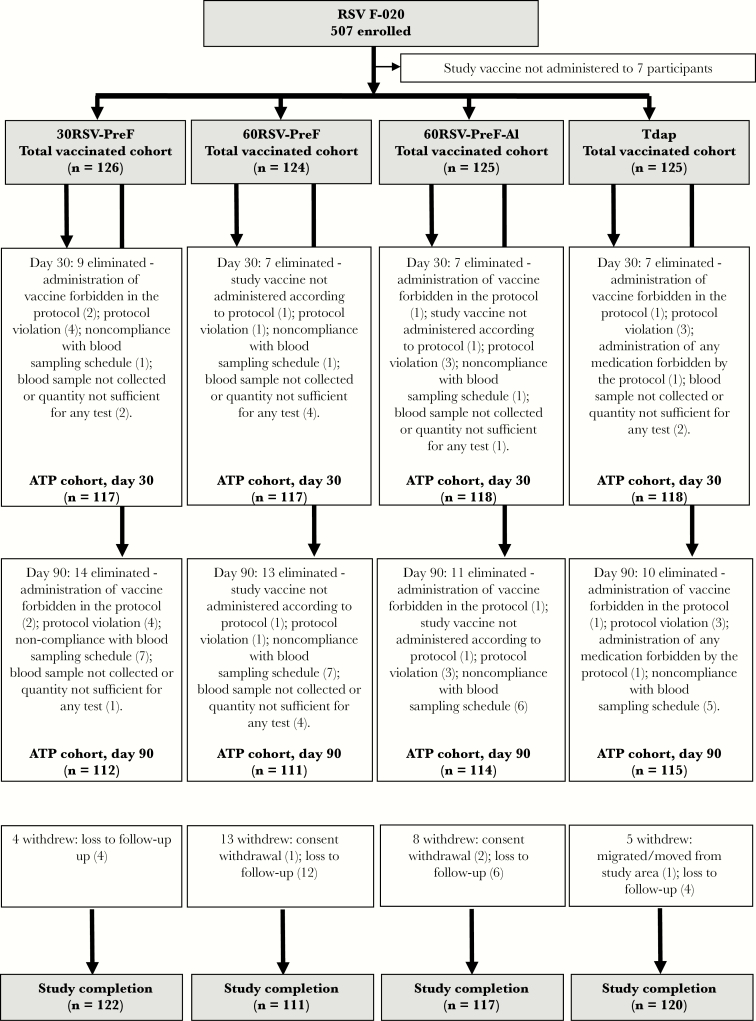
Study flow. The 30RSV-PreF group received nonadjuvanted RSV vaccine containing 30 µg of RSV–prefusion F protein (PreF), the 60RSV-PreF group received nonadjuvanted RSV vaccine containing 60 µg of RSV PreF, the 60RSV-PreF-Al group received aluminum-adjuvanted RSV vaccine containing 60 µg of RSV PreF, and the Tdap group received an adult formulation of combined tetanus toxoid-diphtheria toxoid-acellular pertussis vaccine. ATP, according to protocol.

There were 500 nonpregnant 18–45-year-old women enrolled and vaccinated in RSV F-020 and 100 such women in RSV F-024. There were no withdrawals due to AEs or SAEs (Figure 2). In each study, groups were balanced in terms of demographic characteristics (Table 1).

**Table 1.** Summary of Demographic Characteristics in the Total Vaccinated Cohorts

Characteristic	RSV F-020				RSV F-024	
	30RSV-PreF (n = 126)	60RSV-PreF (n = 124)	60RSV-PreF-Al (n = 125)	Tdap (n = 125)	60RSV-PreF (n = 49)	Tdap (n = 51)
Age at vaccination, y						
Mean ± SD	29.2 ± 7.5	29.5 ± 8.2	29.1 ± 7.4	29.2 ± 7.9	25.8 ± 5.9	25.6 ± 6.1
Range	19–45	18–45	18–45	18–45	19–45	19–43
Geographic ancestry, no. (%)						
African/African American	5 (4.0)	7 (5.6)	9 (7.2)	9 (7.2)	0 (0.0)	0 (0.0)
Asian^a^	3 (2.4)	1 (0.8)	12 (9.6)	4 (3.2)	0 (0.0)	1 (2.0)
White/European	112 (88.9)	114 (91.9)	97 (77.6)	108 (86.4)	49 (100)	50 (98.0)
Other	6 (4.8)	2 (1.6)	7 (5.6)	4 (3.2)	0 (0.0)	0 (0.0)

The 30RSV-PreF group received nonadjuvanted respiratory syncytial virus (RSV) vaccine containing 30 µg of RSV–prefusion F protein (PreF), the 60RSV-PreF groups received nonadjuvanted RSV vaccine containing 60 µg of RSV PreF, the 60RSV-PreF-Al group received aluminum-adjuvanted RSV vaccine containing 60 µg of RSV PreF, and the Tdap groups received an adult formulation of combined tetanus toxoid-diphtheria toxoid-acellular pertussis vaccine.

^a^Participants of Central/South Asian, East Asian, Japanese, or Southeast Asian heritage.

### Safety and Reactogenicity

#### RSV F-020

**Figure 3.**
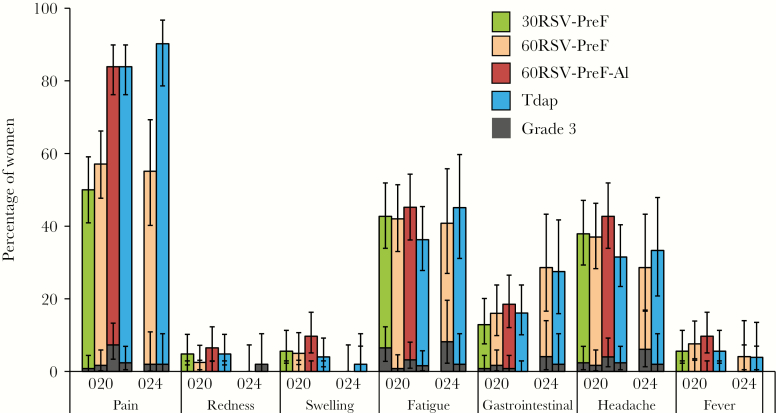
Solicited injection site and general symptoms reported within 7 days (day 0–6) after vaccination in RSV F-020 and F-024. The 30RSV-PreF group received nonadjuvanted RSV vaccine containing 30 µg of RSV–prefusion F protein (PreF), the 60RSV-PreF groups received nonadjuvanted RSV vaccine containing 60 µg of RSV PreF, the 60RSV-PreF-Al group received aluminum-adjuvanted RSV vaccine containing 60 µg of RSV PreF, and the Tdap groups received an adult formulation of combined tetanus toxoid-diphtheria toxoid-acellular pertussis vaccine.

Pain was the most frequently reported solicited local symptom, affecting 50.0% of participants in the 30RSV-PreF group, 57.1% in the 60RSV-PreF group, and 83.9% in the 60RSV-PreF-Al and Tdap groups (Figure 3). Grade 3 pain was reported most frequently in the 60RSV-PreF-Al group (7.3% of participants), compared with 0.8% in the 30RSV-PreF group, 1.7% in the 60RSV-PreF group, and 2.4% in the Tdap group. Redness or swelling was reported by <10% of participants in all groups, and no grade 3 redness or swelling was reported.

The most frequently reported solicited general symptoms in each group were fatigue and headache. Fatigue was reported in 42.7% of participants in the 30RSV-PreF group, 42.0% in the 60RSV-PreF group, 45.2% in the 60RSV-PreF-Al group, and 36.3% in the Tdap group. Grade 3 fatigue was reported in 6.5% of participants in the 30RSV-PreF group, 0.8% in the 60RSV-PreF group, 3.2% in the 60RSV-PreF-Al group, and 1.6% in the Tdap group. The percentage of participants with headache ranged from 37.0% to 37.9% in the nonadjuvanted RSV-PreF groups and was 42.7% in the 60RSV-PreF-Al group and 31.5% in the Tdap group. Grade 3 headache was reported in 1.7%–2.4% of participants in the nonadjuvanted RSV-PreF groups, 4.0% in the 60RSV-PreF-Al group, and 2.4% in the Tdap group.

The percentage of participants with any grade 2/3 AEs, fever (temperature, >38.5°C), and/or any vaccine-related SAE during the 7-day postvaccination period was 31.7% in the 30RSV-PreF group, 27.4% in the 60RSV-PreF group, 45.6% in the 60RSV-PreF-Al group, and 37.6% in the Tdap group. Exploratory comparisons for reactogenicity end points (defined as the percentage of subjects reporting any grade 2/3 adverse events and/or fever [temperature, >38.5°C]) indicated a statistically significantly higher incidence in the 60RSV-PreF-Al group as compared to the 30RSV-PreF and 60RSV-PreF groups (Supplementary Table 1).

The rates of unsolicited AEs were comparable among all groups. The percentage of participants who reported unsolicited symptoms until 30 days after vaccination was 27.8% in the 30RSV-PreF group, 30.6% in the 60RSV-PreF group, 27.2% in the 60RSV-PreF-Al group, and 29.6% in the Tdap group. Unsolicited AEs considered by the investigator to be related to vaccination were reported by 7.1% of participants in the 30RSV-PreF group, 12.1% in the 60RSV-PreF group, 10.4% in the 60RSV-PreF-Al group, and 9.6% in the Tdap group. Vaccine-related events reported by >2 participants in any group were influenza-like illness (3 participants in the 60RSV-PreF group), injection site pruritus (3 participants in the 60RSV-PreF-Al group), and myalgia (3 participants in the 60RSV-PreF group). One grade 3 AE (“pain in extremity,” reported in the 30RSV-PreF group) was considered to be related to vaccination. There were 16 SAEs (all nonfatal) reported from vaccination until day 360 (Supplementary Table 2). One SAE (constrictive bronchiolitis, reported in the 30RSV-PreF group, with onset 38 days after vaccination in a previously healthy woman), was initially considered potentially related to vaccination by the investigator, but after receipt of anti-N titers the investigator changed his assessment to “not related to vaccination.” The SAE was considered not related to vaccination by the independent data monitoring committee, the study sponsor, and an external pulmonary consultant. A research assay able to detect serological responses against the RSV N protein following natural RSV infection was developed by the study sponsor. The N protein is expressed during RSV infection but not contained in this investigational vaccine. No increase in anti-N titer was observed between days 0 and 30, days 0 and 60, or days 0 and 90 in the subject with constrictive bronchiolitis, suggesting that an RSV infection following vaccination did not contribute to the pulmonary disease observed at day 38. Lung histopathologic findings were suggestive of pneumoconiosis, possibly related to occupational/cosmetologist exposure to microdermabrasion and silica/inert crystals and complicated by *Pseudomonas aeruginosa* infection, although the subject had no clinical signs or symptoms of infection. The histopathologic findings were consistent with a preexisting chronic lung disease.

Fourteen pregnancies in 13 women (1 participant became pregnant again shortly after a spontaneous abortion) were reported during the study, all in RSV F recipients. Outcomes of 9 pregnancies were recorded as live births, 2 as induced abortions, and 2 as spontaneous abortions (1 in the 60RSV-PreF group at 10 weeks of gestation and 1 in the 60RSV-PreF-Al group at 7 weeks of gestation; Supplementary Table 3). One participant was lost to follow-up. All 9 live births occurred at term and involved delivery of healthy infants (Supplementary Table 3). No apparent congenital anomaly was reported in any pregnancy. Women associated with 5 live births had had their last menstrual period during or around the time of the RSV transmission season. The time between exposure and estimated conception was approximately 36 weeks in both participants who experienced spontaneous abortion, and both abortions occurred toward the end of the RSV transmission season. The site investigator considered that there was no reasonable possibility that the event may have been caused by the investigational vaccine.

#### RSV F-024

The reactogenicity profile for 60RSV-PreF within 7 days after vaccination in RSV F-024 was consistent with observations in RSV F-020 (Figure 3). Rates of local and general solicited AEs were comparable between the 60RSV-PreF and Tdap groups. The percentage of participants who reported unsolicited symptoms until 30 days after vaccination was 46.9% in the 60RSV-PreF group, compared with 54.9% in the Tdap group. The percentage of participants with unsolicited AEs considered by the investigator to be related to vaccination was 24.5% in the 60RSV-PreF group and 19.6% in the Tdap group. The most frequently reported vaccine-related AEs were fatigue (reported by 3 participants in the Tdap group), myalgia (2 each in the 60RSV-PreF and Tdap groups), dizziness (3 in the 60RSV-PreF group), and oropharyngeal pain and rash (2 in the Tdap group for each AE). Six subjects reported the following 7 grade 3 vaccine-related AEs: arthralgia, headache, and respiratory disorder in the 60RSV-PreF group and fatigue, myalgia, oropharyngeal pain, and tonsillar disorder in the Tdap group. There were no SAEs or pregnancies reported during the 30-day study follow-up period.

#### Clinical Laboratory Evaluations (RSV F-024)

The majority of hematologic and biochemical parameters measured on days 7 and 30 remained unchanged throughout the study period. There was 1 grade 2 decrease in neutrophil count in the 60RSV-PreF group and 1 grade 2 decrease in lymphocyte count in the Tdap group following vaccination. There was 1 case of grade 4 anemia in the Tdap group; this participant had low hemoglobin level on day 0, before vaccination (FDA toxicity grade 4; results were available only after vaccination had occurred). Hemoglobin values improved slightly by day 30 (FDA toxicity grade 3). An etiology was not determined.

### Immunogenicity (RSV F-020)

#### Neutralizing Antibodies

**Figure 4.**
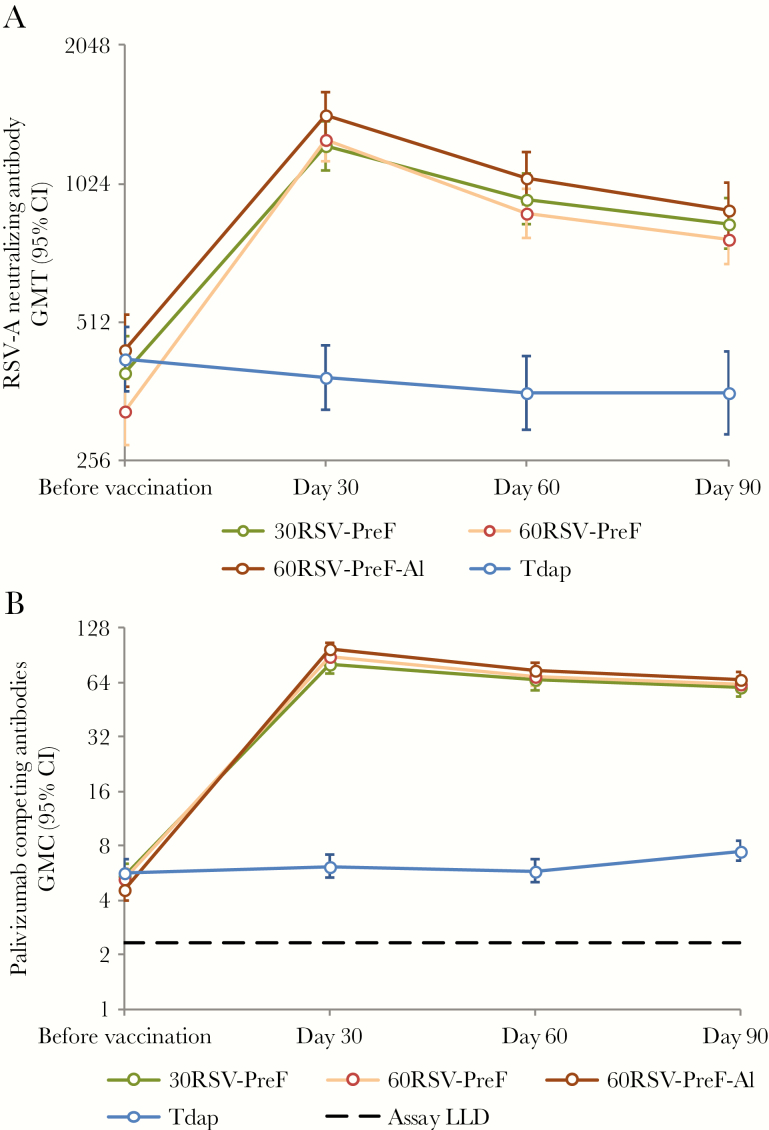
(A) Geometric mean anti–respiratory syncytial virus subtype A (RSV-A) neutralizing antibody titers and (B) geometric mean palivizumab-competing antibody concentrations with 95% confidence intervals until day 90 after vaccination—RSV F-020, according-to-protocol immunogenicity cohort. The 30RSV-PreF group received nonadjuvanted RSV vaccine containing 30 µg of RSV–prefusion F protein (PreF), the 60RSV-PreF group received nonadjuvanted RSV vaccine containing 60 µg of RSV PreF, the 60RSV-PreF-Al group received aluminum-adjuvanted RSV vaccine containing 60 µg of RSV PreF, and the Tdap group received an adult formulation of combined tetanus toxoid-diphtheria toxoid-acellular pertussis vaccine. LLD, lower limit of detection.

All subjects were seropositive for RSV-A neutralizing antibodies at baseline (Figure 4). In all RSV-PreF groups, preexisting RSV-A neutralizing antibody responses were boosted by a single dose of RSV-PreF vaccine (Figure 4). The percentage of RSV-PreF vaccinees with RSV-A neutralizing titers of ≥1024 was 61.5%–71.2% among the groups on day 30, 37.8%–52.8% on day 60, and 34.2%–43.6% on day 90 (Table 2). Neutralizing antibody vaccine response rates (see Table 2 for definition of a vaccine response) were observed in 37.6%– 77.1% of RSV-PreF vaccinees, compared with ≤5.1% of Tdap vaccinees (Table 2).

**Table 2.** Respiratory Syncytial Virus Subtype A (RSV-A) Neutralizing Antibody Titers—RSV F-020, According-to-Protocol Cohort for Immunogenicity

Group,^a^ Timing	Evaluated, No.	Titer, No. (%)		Vaccine Response^b^	
		≥512	≥1024	Evaluated, No.	Response, No. (%)
30RSV-PreF					
Before vaccination	117	43 (36.8)	18 (15.4)	…	…
After vaccination					
Day 30	117	107 (91.5)	74 (63.2)	117	81 (69.2)
Day 60	109	89 (81.7)	49 (45.0)	108	52 (48.1)
Day 90	112	83 (74.1)	43 (38.4)	111	42 (37.8)
60RSV-PreF					
Before vaccination	117	40 (34.2)	12 (10.3)	…	…
After vaccination					
Day 30	117	110 (94.0)	72 (61.5)	117	83 (70.9)
Day 60	111	90 (81.1)	42 (37.8)	111	59 (53.2)
Day 90	111	78 (70.3)	38 (34.2)	111	42 (37.8)
60RSV-PreF-Al					
Before vaccination	118	50 (42.4)	26 (22.0)	…	…
After vaccination					
Day 30	118	111 (94.1)	84 (71.2)	118	91 (77.1)
Day 60	108	93 (86.1)	57 (52.8)	108	56 (51.9)
Day 90	110	86 (78.2)	48 (43.6)	110	49 (44.5)
Tdap					
Before vaccination	118	45 (38.1)	20 (16.9)	…	…
After vaccination					
Day 30	118	42 (35.6)	17 (14.4)	118	6 (5.1)
Day 60	111	39 (35.1)	14 (12.6)	109	5 (4.6)
Day 90	111	45 (40.5)	14 (12.6)	109	4 (3.7)

^a^The 30RSV-PreF group received nonadjuvanted RSV vaccine containing 30 µg of RSV–prefusion F protein (PreF), the 60RSV-PreF group received nonadjuvanted RSV vaccine containing 60 µg of RSV PreF, the 60RSV-PreF-Al group received aluminum-adjuvanted RSV vaccine containing 60 µg of RSV PreF, and the Tdap group received an adult formulation of combined tetanus toxoid-diphtheria toxoid-acellular pertussis vaccine.

^b^Vaccine response rates involving neutralizing antibodies were calculated as follows: at least a 4-fold increase from the prevaccination level if the prevaccination neutralizing antibody titer was <128, at least a 3-fold increase from the prevaccination level if the prevaccination neutralizing antibody titer was [128–256], at least a 2.5-fold increase from the prevaccination level if the prevaccination neutralizing antibody titer was [256–1024], and at least a 1-fold increase from the prevaccination level if the prevaccination neutralizing antibody titer was >1024.

In RSV-PreF vaccinees, RSV-A neutralizing antibody GMTs increased 3.1–3.9-fold by day 30, compared with prevaccination titers (Table 3). By day 90, RSV-A neutralizing antibodies had declined in all RSV-PreF groups but remained at least 2.0-fold higher than prevaccination values (Table 3). Exploratory comparisons suggested no difference in RSV-A neutralizing antibody levels between the 3 RSV-PreF groups 30 days after vaccination (Supplementary Table 4). The kinetics of the RSV-A neutralizing antibody response appeared similar in all 3 RSV groups (Figure 4).

**Table 3.** Ratios of Geometric Mean Titers (GMTs) and Geometric Mean Antibody Concentrations (GMCs) on Days 30, 60, and 90 After Vaccination to Values Before Vaccination—RSV F-020, According-to-Protocol Immunogenicity Cohort

Group,^a^ Time Point	Evaluated, No.	Postvaccination Value	Prevaccination Value	Ratio of Values (95% CI)
Geometric mean RSV subtype A neutralizing antibody titers				
30RSV-PreF				
Day 30	117	1237.0	399.4	3.1 (2.7–3.6)
Day 60	108	958.1	401.4	2.4 (2.1–2.8)
Day 90	111	843.0	397.2	2.1 (1.9–2.4)
60RSV-PreF				
Day 30	117	1278.7	326.3	3.9 (3.4–4.6)
Day 60	111	882.9	319.5	2.8 (2.4–3.2)
Day 90	111	774.5	319.5	2.4 (2.1–2.8)
60RSV-PreF-Al				
Day 30	118	1442.5	446.8	3.2 (2.8–3.7)
Day 60	108	1055.7	461.4	2.3 (2.0–2.7)
Day 90	110	897.5	452.8	2.0 (1.7–2.3)
Tdap				
Day 30	118	387.1	423.7	0.9 (.9–1.0)
Day 60	109	367.9	432.5	0.9 (.8–.9)
Day 90	109	368.8	440.4	0.8 (.8–.9)
Geometric mean palivizumab-competing antibody concentrations				
30RSV-PreF				
Day 30	108	79.5	5.6	14.1 (12.0–16.6)
Day 60	103	65.7	5.7	11.5 (9.7–13.6)
Day 90	106	60.0	5.5	10.8 (9.3–12.6)
60RSV-PreF				
Day 30	106	86.9	5.1	16.9 (14.1–20.3)
Day 60	101	68.2	5.1	13.4 (11.3–16.0)
Day 90	101	62.3	5.1	12.2 (10.4–14.4)
60RSV-PreF-Al				
Day 30	103	97.8	4.7	21.0 (18.0–24.5)
Day 60	96	75.0	4.5	16.5 (13.9–19.6)
Day 90	97	66.2	4.6	14.2 (12.2–16.7)
Tdap				
Day 30	105	6.4	6.0	1.1 (1.0–1.2)
Day 60	102	6.2	5.9	1.1 (1.0–1.1)
Day 90	101	7.9	6.0	1.3 (1.2–1.4)

Abbreviations: CI, confidence interval; RSV, respiratory syncytial virus.

^a^The 30RSV-PreF group received nonadjuvanted RSV vaccine containing 30 µg of RSV–prefusion F protein (PreF), the 60RSV-PreF group received nonadjuvanted RSV vaccine containing 60 µg of RSV PreF, the 60RSV-PreF-Al group received aluminum-adjuvanted RSV vaccine containing 60 µg of RSV PreF, and the Tdap group received an adult formulation of combined tetanus toxoid-diphtheria toxoid-acellular pertussis vaccine.

#### IgG Subclass Analysis by ELISA

Before vaccination, all participants were RSV seropositive when tested for total IgG antibody. Almost all (≥97.9%) were seropositive for anti-RSV-F IgG subclass 1. Vaccination induced 25.7–38.2-fold increases in total anti-RSV IgG antibodies and 15.7–20.1-fold increases in IgG subclass 1 antibodies in the RSV-PreF groups on day 30. No increases were observed in the control group (Supplementary Table 5). Exploratory comparisons suggested no differences between RSV-PreF groups in the magnitude of the fold increase in IgG1 or total IgG levels on day 30 (data not shown).

#### Palivizumab-Competing Antibodies

Before vaccination, 72.4%–78.5% of participants were seropositive for PCAs, although GMCs were close to the assay cutoff (Figure 4). Postvaccination PCA concentrations were 14.1–21.0-fold higher on day 30 than at baseline in each RSV group (Table 3). By day 90, PCA levels declined in each RSV group but remained >10-fold higher than prevaccination levels (Table 2).

Exploratory comparisons indicated that the PCA GMC on day 30 was higher in participants in the 60RSV-PreF-Al group, compared with that in the 30RSV-PreF group (GMC ratio, 1.25; 95% CI, 1.05–1.49). No other group comparisons were statistically significant (Supplementary Table 5). The kinetics of the PCA immune response appeared similar in all 3 RSV groups (Figure 4).

## DISCUSSION

The 2 studies presented here confirm and expand the findings of the phase 1 study conducted in men, which showed that RSV-PreF vaccine boosted humoral immune responses to RSV, with a favorable reactogenicity and safety profile [13]. We built on these results in a larger cohort of women of childbearing age. In both studies, the nonadjuvanted RSV-PreF candidate vaccines were less reactogenic (especially in terms of injection site pain) than Tdap, a vaccine that is already considered the standard of care for use during pregnancy in many countries. Exploratory analyses showed a trend for higher reactogenicity in the 60RSV-PreF-Al group, compared with the nonadjuvanted RSV-PreF groups, without evidence of an immunological benefit of the adjuvant. Based on these findings, the nonadjuvanted 60RSV-PreF vaccine was selected for study RSV F-024, which assessed reactogenicity, safety, biochemical, and hematological parameters after vaccination with 60RSV-PreF.

No safety concerns were identified that would preclude continued or future development for any of the RSV-PreF groups in either study, and the safety profile of the vaccine was similar to that of Tdap. In the final analysis, none of the SAEs were considered by the investigators to be related to vaccination. Two spontaneous abortions were reported for 2 women in the 60RSV-PreF group in RSV F-020. Both women conceived >7 months after vaccination, and the abortions were assessed as not related to vaccination by the investigator. The frequency of abnormalities among biochemical and hematologic parameters after vaccination was low and comparable between study groups in RSV F-024.

All RSV-PreF vaccine formulations boosted RSV-A neutralizing antibody titers, with many subjects reaching the cutoff of 1024, selected because of evidence suggesting that this level may be meaningful in providing protection until 4 months after delivery [15, 16]. An adjuvant effect was not discernable. PCA concentrations also increased markedly, suggesting binding of vaccine-induced antibodies to the antigenic site II of the F protein. Levels of RSV-PreF total IgG and IgG1, which is the antibody subclass that is most efficiently transferred across the placenta [17], increased substantially from baseline to day 30 after vaccination.

There were 424 healthy women of childbearing age who received an RSV-PreF vaccine formulation in the 2 studies presented here. A potential limitation of both studies is that the appearance of the study vaccines differed from that of Tdap, which precluded a double-blinded study design. However, the observer-blinded design should have avoided any observer bias. Potential confounding of immune responses due to natural boosting that might occur if any of the subjects developed an RSV infection during the study was not assessed, but any impact of this was largely overcome by the controlled study design and by enrolling most subjects outside the RSV transmission season.

In conclusion, all 3 formulations of an experimental RSV-PreF vaccine boosted preexisting immune responses in 18–45-year-old women, with comparable immunogenicity. The RSV-PreF safety profile was similar to that of Tdap.

## Supplementary Data

Supplementary materials are available at *The Journal of Infectious Diseases* online. A plain language summary contextualizing the results and potential clinical research relevance and impact is displayed in the Focus on Patient Section available in the supplementary materials. Consisting of data provided by the authors to benefit the reader, the posted materials are not copyedited and are the sole responsibility of the authors, so questions or comments should be addressed to the corresponding author.

Supplementary Table 1

Supplementary Table 2

Supplementary Table 3

Supplementary Table 4

Supplementary Table 5

Supplementary Material

## Notes

***Author contributions.***  T.F.S., L.A.F.,M.P.D., K.M., M.P. and I.D. conceived and designed the study. J.B., J.D.L., T.F.S., C.J., L.C., J.B.D., P.V.D., K.W., K.M., A.C.S., M.P. and I.D. collected or generated study data. J.B., J.D.L., T.F.S., C.J., J.B.D., P.V.D., K.W., K.M., A.C.S., M.P. and I.D. performed the study. J.D.L., T.F.S., J.B.D., M.P.D., K.M. and I.D. contributed materials/analysis/reagent tools. J.B., J.D.L., J.B.D., P.V.D., L.A.F., M.P.D., K.M., A.C.S., M.P. and I.D. were involved in the analyses and/or interpretation of the data. All authors contributed to the writing and review of the manuscript and approved the final version for submission.

***Acknowledgments.***  Acknowledgments are available in the Supplementary Materials.

***Financial support.*** This work was supported by GSK Biologicals SA, which was involved in all stages of the study conduct and analysis, and also took responsibility for all costs associated with the development and publishing of the present manuscript.

***Potential conflicts of interest.*** J. B. reports receiving grants from GSK, based on the study budget, as agreed by the European Commission, for performing this clinical trial at the center in the Czech Republic. J. D. L. reports receiving grants from GSK during the conduct of the study. T. F. S. reports receiving personal fees from the GSK group of companies, Sanofi Pasteur, and Pfizer outside the submitted work. J. B. D. reports receiving grants from the GSK group of companies during the conduct of the study and from Pfizer, AstraZeneca, and Regeneron. P. V. D. and K. W. act as investigators for vaccine trials conducted on behalf of the University of Antwerp, for which the university obtained research grants from GSK during the conduct of the study and from other vaccine companies outside the submitted work. L. A. F., M.-P. D., K. M., A. C. S., M. P., and I. D. are employees of the GSK group of companies. M.-P. D., K. M., A. C. S., M. P., and I. D. hold shares or stock options in the GSK group of companies as part of their employee remuneration. All other authors report no potential conflicts of interest. All authors have submitted the ICMJE Form for Disclosure of Potential Conflicts of Interest. Conflicts that the editors consider relevant to the content of the manuscript have been disclosed.

Presented in part: 10th International Respiratory Syncytial Virus Symposium, Patagonia, Argentina, 28 September-1 October 2016.
