# Supplementary material for: Safety and Immunogenicity of 3 Formulations of an Investigational Respiratory Syncytial Virus Vaccine in Nonpregnant Women: Results From 2 Phase 2 Trials
Source: J Infect Dis. 2018 Feb 1;217(10):1616–25. doi: 10.1093/infdis/jiy065 (PMC5913599; doi:10.1093/infdis/jiy065)
Supplement: Supplementary Table 1 [file jiy065_suppl_supplementary_table_1.docx]

**Supplementary Table 1:** Exploratory comparisons between groups in terms of percentage of subjects reporting any Grade 2/3 adverse events and/or any fever >38.5°C and/or any vaccine-related Serious Adverse Events during the 7 day (Days 0-6) post-vaccination period (RSV F-020, total vaccinated cohort)

| **Group 1** | **N** | **n** | **%** | **Group 2** | **N** | **n** | **%** | **Difference (Group 1 minus Group 2)** | **% (95% CI)** |
| --- | --- | --- | --- | --- | --- | --- | --- | --- | --- |
| 30RSV-PreF | 126 | 40 | 31.7 | Tdap | 125 | 47 | 37.6 | 30RSV-PreF - Tdap | -5.85 (-17.51; 5.94) |
| 60RSV-PreF | 124 | 34 | 27.4 | Tdap | 125 | 47 | 37.6 | 60RSV-PreF - Tdap | -10.18 (-21.61; 1.49) |
| 60RSV-PreF-Al | 125 | 57 | 45.6 | Tdap | 125 | 47 | 37.6 | 60RSV-PreF-Al - Tdap | 8.00 (-4.24; 20.01) |
| 60RSV-PreF | 124 | 34 | 27.4 | 30RSV-PreF | 126 | 40 | 31.7 | 60RSV-PreF - 30RSV-PreF | -4.33 (-15.58; 7.03) |
| 60RSV-PreF-Al | 125 | 57 | 45.6 | 30RSV-PreF | 126 | 40 | 31.7 | 60RSV-PreF-Al - 30RSV-PreF | 13.85 (1.79; 25.55) |
| 60RSV-PreF-Al | 125 | 57 | 45.6 | 60RSV-PreF | 124 | 34 | 27.4 | 60RSV-PreF-Al - 60RSV-PreF | 18.18 (6.24; 29.65) |

N = number of participants with the administered dose

n (%) = number (percentage) of participants reporting a specified symptom

95% CI = standardized asymptotic

30RSV-PreF = non-adjuvanted RSV vaccine containing 30µg PreF, 60RSV-PreF = non-adjuvanted RSV vaccine containing 60µg PreF, 60RSV-PreF-Al = aluminum-adjuvanted RSV vaccine containing 60µg PreF, Tdap = combined tetanus-diphtheria-acellular pertussis vaccine
