# Supplementary material for: Safety and Immunogenicity of 3 Formulations of an Investigational Respiratory Syncytial Virus Vaccine in Nonpregnant Women: Results From 2 Phase 2 Trials
Source: J Infect Dis. 2018 Feb 1;217(10):1616–25. doi: 10.1093/infdis/jiy065 (PMC5913599; doi:10.1093/infdis/jiy065)
Supplement: Supplementary Table 2 [file jiy065_suppl_supplementary_table_2.docx]

**Supplementary Table 2**: Listing of SAEs reported up to study end (Total vaccinated cohort)

| **Age (years)** | **Verbatim** | **Preferred term** | **Primary System Organ Class** | **Day of onset** | **Duration (days)** | **Outcome** |
| --- | --- | --- | --- | --- | --- | --- |
| **30RSV-PreF** | | | | | | |
| 31 | Left maxillary cyst | Jaw cyst | Musculoskeletal and connective tissue disorders | 2 | 119 | Recovered/resolved |
| 39 | Constrictive bronchiolitis | Obliterative bronchiolitis | Respiratory, thoracic and mediastinal disorders | 38 | . | Not recovered/not resolved |
| 19 | Suicide attempt | Suicide attempt | Psychiatric disorders | 69 | 10 | Recovered/resolved |
| **60RSV-PreF** | | | | | | |
| 20 | Musculoskeletal injuries | Musculoskeletal injury | Injury, poisoning and procedural complications | 8 | . | Recovering/resolving |
| 24 | Spontaneous abortion | Abortion spontaneous | Pregnancy, puerperium and perinatal conditions | 310 | 1 | Recovered/resolved |
| 43 | Transient ischemic attack | Transient ischaemic attack | Nervous system disorders | 85 | 4 | Recovered/resolved |
| **60RSV-PreF-Al** | | | | | | |
| 37 | Umbilical hernia | Umbilical hernia | Gastrointestinal disorders | 125 | 40 | Recovered/resolved |
| 22 | Virally induced asthma | Asthma | Respiratory, thoracic and mediastinal disorders | 77 | 7 | Recovered/resolved |
| 46 | Colitis | Colitis | Gastrointestinal disorders | 215 | 5 | Recovered/resolved |
| 46 | Incisional hernia | Incisional hernia | Injury, poisoning and procedural complications | 235 | 1 | Recovered/resolved |
| 30 | Spontaneous abortion | Abortion spontaneous | Pregnancy, puerperium and perinatal conditions | 289 | 1 | Recovered/resolved |
| 27 | Weber b fracture right ankle | Fibula fracture | Injury, poisoning and procedural complications | 264 | 11 | Recovered/resolved |
| 27 | Lateral tibia head fracture right leg | Tibia fracture | Injury, poisoning and procedural complications | 264 | 11 | Recovered/resolved |
| 30 | Erysipelas cheek | Erysipelas | Infections and infestations | 318 | 70 | Recovered/resolved |
| **Tdap** |  |  |  |  |  |  |
| 27 | Polytrauma | Multiple injuries | Injury, poisoning and procedural complications | 213 | 62 | Recovered/resolved with sequelae |
| 33 | Bacterial pneumonia | Pneumonia bacterial | Infections and infestations | 68 | 19 | Recovered/resolved |

30RSV-PreF = non-adjuvanted RSV vaccine containing 30µg PreF, 60RSV-PreF = non-adjuvanted RSV vaccine containing 60µg PreF, 60RSV-PreF-Al = aluminum-adjuvanted RSV vaccine containing 60µg PreF, Tdap = combined tetanus-diphtheria-acellular pertussis vaccine
