# Supplementary material for: Safety and Immunogenicity of 3 Formulations of an Investigational Respiratory Syncytial Virus Vaccine in Nonpregnant Women: Results From 2 Phase 2 Trials
Source: J Infect Dis. 2018 Feb 1;217(10):1616–25. doi: 10.1093/infdis/jiy065 (PMC5913599; doi:10.1093/infdis/jiy065)
Supplement: Supplementary Table 3 [file jiy065_suppl_supplementary_table_3.docx]

**Supplementary Table 3**: Pregnancy outcomes in participants vaccinated in study RSV-F-020

| **Country** | **LMP** | **Outcome** | **Gestation** | **Apgar** | **Birth weight** |
| --- | --- | --- | --- | --- | --- |
| **30RSV-PreF** |  |  |  |  |  |
| Czech Republic | 16-Feb-16 | Live birth | 40 weeks | 10/10/10 | 3850g |
| United States | 10-Jul-15 | Live birth | 40+ weeks | 9/10/10 | 4240g |
| Czech Republic | 14-Oct-15 | Live birth | 38+ weeks | 9/10/10 | 3410g |
| Czech Republic | 09-Jul-15 | Live birth | 38+ weeks | 10/10/10 | 2700g |
| Australia | 09-Aug-15 | Live birth | 40+ weeks | Not known | Not known |
| Germany | 16-Jul-15 | Live birth | 42 weeks | 10/10/10 | 3700g |
| Germany | 17-Jun-15 | Live birth | 40+ weeks | 10/10/10 | 3560g |
| **60RSV-PreF** |  |  |  |  |  |
| Australia | 23-Oct-15 | Induced abortion | 6+ weeks | - | - |
| Australia | 26-Dec-15* | Induced abortion | 9+ weeks* | - | - |
| Germany | 02-Nov-15 | Live birth | 39 weeks | 10/10/10 | 3220g |
| Germany | 23-Dec-15 | Spontaneous abortion | 10 weeks | - | - |
| **60RSV-PreF-Al** |  |  |  |  |  |
| Germany | 09-Dec-15 | Lost to follow-up | - | - | - |
| Germany | 23-Dec-15 | Spontaneous abortion | 7 weeks | - | - |
| Germany | 09-Feb-16 | Live birth | 40 weeks | 10/10/10 | 3190g |

* LMP and gestation were calculated based on partial dates provided for this subject

30RSV-PreF = non-adjuvanted RSV vaccine containing 30µg PreF, 60RSV-PreF = non-adjuvanted RSV vaccine containing 60µg PreF, 60RSV-PreF-Al = aluminum-adjuvanted RSV vaccine containing 60µg PreF, Tdap = combined tetanus-diphtheria-acellular pertussis vaccine

LMP = date of the last menstrual period
