# Supplementary material for: Safety and Immunogenicity of 3 Formulations of an Investigational Respiratory Syncytial Virus Vaccine in Nonpregnant Women: Results From 2 Phase 2 Trials
Source: J Infect Dis. 2018 Feb 1;217(10):1616–25. doi: 10.1093/infdis/jiy065 (PMC5913599; doi:10.1093/infdis/jiy065)
Supplement: Supplementary Table 4 [file jiy065_suppl_supplementary_table_4.docx]

**Supplementary Table 4:** Exploratory comparisons (GMT/C ratios) between RSV-PreF groups with corresponding 95% confidence intervals* for anti-RSV-A neutralizing antibody titers and Palivizumab competing antibody concentrations at Day 30 (RSV F-020, according-to-protocol immunogenicity cohort)

| **Group** | **N** | **GMT/C** | **Group** | **N** | **GMT/C** | **Ratio order** | **Ratio (95% CI)** |
| --- | --- | --- | --- | --- | --- | --- | --- |
| **Anti-RSV-A neutralizing antibody** | | | | | | | |
| 60RSV-PreF-Al | 118 | 1371.5 | 60RSV-PreF | 117 | 1358.9 | 60RSV-PreF- Al/60RSV-PreF | 1.01 (0.86; 1.19) |
| 60RSV-PreF- Al | 118 | 1371.5 | 30RSV-PreF | 117 | 1224.7 | 60RSV-PreF-Al/30RSV-PreF | 1.12 (0.95; 1.32) |
| 60RSV-PreF | 117 | 1358.9 | 30RSV-PreF | 117 | 1224.7 | 60RSV-PreF/30RSV-PreF | 1.11 (0.94; 1.31) |
| **Palivizumab competing antibody** | | | | | | | |
| 60RSV-PreF-Al | 105 | 98.6 | 60RSV-PreF | 107 | 86.8 | 60RSV-PreF- Al/60RSV-PreF | 1.14 (0.95; 1.35) |
| 60RSV-PreF- Al | 105 | 98.6 | 30RSV-PreF | 110 | 79.0 | 60RSV-PreF-Al/30RSV-PreF | 1.25 (1.05; 1.49) |
| 60RSV-PreF | 107 | 86.8 | 30RSV-PreF | 110 | 79.0 | 60RSV-PreF/30RSV-PreF | 1.10 (0.92; 1.31) |

* Tukey’s 95% confidence interval for the GMT/C ratio (ANCOVA model, Tukey’s adjustment)

GMT/C = geometric mean antibody titer/concentration

N = number of subjects with available results at the specified time point

30RSV-PreF = non-adjuvanted RSV vaccine containing 30µg PreF, 60RSV-PreF = non-adjuvanted RSV vaccine containing 60µg PreF, 60RSV-PreF-Al = aluminum-adjuvanted RSV vaccine containing 60µg PreF, Tdap = combined tetanus-diphtheria-acellular pertussis vaccine
