# Supplementary material for: Safety and Immunogenicity of 3 Formulations of an Investigational Respiratory Syncytial Virus Vaccine in Nonpregnant Women: Results From 2 Phase 2 Trials
Source: J Infect Dis. 2018 Feb 1;217(10):1616–25. doi: 10.1093/infdis/jiy065 (PMC5913599; doi:10.1093/infdis/jiy065)
Supplement: Supplementary Table 5 [file jiy065_suppl_supplementary_table_5.docx]

**Supplementary Table 5:** Geometric mean of the individual ratio of total anti-RSV F IgG and IgG subclass 1 antibody titers at Day 30 compared to pre-vaccination (RSV F-020, according-to-protocol immunogenicity cohort)

| **Group** | **N** | **GMT at**  **Day 30** | **GMT before**  **vaccination** | **Ratio (95% CI)** |
| --- | --- | --- | --- | --- |
| **IgG subclass 1** |  |  |  |  |
| 30RSV-PreF | 48 | 2515.0 | 160.3 | 15.7 (12.7; 19.4) |
| 60RSV-PreF | 49 | 3336.3 | 208.8 | 16.0 (12.7; 20.1) |
| 60RSV-PreF-Al | 47 | 3212.6 | 159.4 | 20.1 (16.6; 24.5) |
| Tdap | 48 | 210.6 | 223.9 | 0.9 (0.9; 1.0) |
| **Total IgG** |  |  |  |  |
| 30RSV-PreF | 48 | 52,493.7 | 1862.5 | 28.2 (21.3; 37.3) |
| 60RSV-PreF | 49 | 69,830.3 | 2712.0 | 25.7 (19.4; 34.1) |
| 60RSV-PreF-Al | 47 | 69,653.9 | 1825.7 | 38.2 (30.7; 47.4) |
| Tdap | 48 | 2813.7 | 2872.0 | 1.0 (0.9; 1.0) |

GMT = geometric mean antibody titer

N = number of subjects with available results at the two considered time points

95% CI = 95% confidence interval

30RSV-PreF = non-adjuvanted RSV vaccine containing 30µg PreF, 60RSV-PreF = non-adjuvanted RSV vaccine containing 60µg PreF, 60RSV-PreF-Al = aluminum-adjuvanted RSV vaccine containing 60µg PreF, Tdap = combined tetanus-diphtheria-acellular pertussis vaccine
