# Supplementary material for: Safety and Immunogenicity of 3 Formulations of an Investigational Respiratory Syncytial Virus Vaccine in Nonpregnant Women: Results From 2 Phase 2 Trials
Source: J Infect Dis. 2018 Feb 1;217(10):1616–25. doi: 10.1093/infdis/jiy065 (PMC5913599; doi:10.1093/infdis/jiy065)
Supplement: Supplementary Material [file jiy065_suppl_supplementary_material.docx]

**Supplementary Materials**

**Focus on the Patient**

**What is the context?**

Respiratory syncytial virus (RSV) is the most common viral cause of severe acute respiratory illness in the first year of life. Young infants are at highest risk for severe RSV disease, including bronchiolitis and pneumonia, and the majority of RSV-related hospitalizations occur during the first 3 months of life. Maternal immunization during the third trimester of pregnancy aims to protect the infant for the first months of life.

**What is new?**

Two phase II clinical trials assessed the safety and immunogenicity of an RSV candidate vaccine in young non-pregnant women. All formulations investigated were able to boost RSV antibody titers and their safety profile was similar to Tdap, a vaccine now recommended during pregnancy in many parts of the world.

**What is the impact?**

Both studies indicate that this investigational RSV vaccine can significantly boost humoral immune responses to RSV in women of childbearing age and was well tolerated. Vaccination of pregnant women could potentially protect infants early in life.

**Methods - Exclusion criteria**

Women were excluded from either study if they were immunocompromised, had a family history of immunodeﬁciency, had autoimmune disease, any malignancy within the last 5 years, a history of hypersensitivity to latex or any vaccine component, acute illness or fever, if they were participating in another clinical study, had received or intended to receive another vaccine within 30 days previous to or after the study vaccine (with the exception of inﬂuenza vaccine, which could be administered ≥15 days before study vaccination), or if they had received either immunoglobulins or blood products, including any immunoglobulin preparation within the last 3 months, or previous RSV vaccination or any investigational product within 30 days prior to vaccination. Participants in RSV F-020 with BMI>45 kg/m^2^ were eliminated from the according-to-protocol analysis. Women were excluded from RSV F-024 if their body mass index was >40 kg/m^2^.

**Acknowledgements**

The authors thank the women who participated in these studies, the many study teams who contributed, as well as the clinical investigators involved in the studies. The authors are grateful for the contribution of Ilse De Coster (University of Antwerp, Antwerp, Belgium) involved in RSV F-024, Rongman Cai and Feng Gao (GSK, Rockville, MD, US) from the statistical team for RSV F-020, Valentina Attanasi and Andrea Sutherland (GSK, Rockville, MD, US) from the Safety team for RSV F-024, Myriam Acosta, Thi Lien Anh Nguyen and Lena Schütte from the Clinical Laboratory Sciences team, Isabelle Labie (GSK, Rixensart, Belgium) from the Data Management team, and Mohamed Bassyouni (GSK, Rockville, MD, US), Geneviève Meiers (GSK, Rixensart, Belgium), Ingrid Deldinne, Valérie Sengers, Valérie Tielemans (GSK, Wavre, Belgium) from the Clinical Operations team for both studies.

Medical writing services were provided by Joanne Wolter (Independent medical writer on behalf of GSK) and Lucia Adina Truta (XPE Pharma & Science on behalf of GSK). Editing and publication coordination services were provided by Quentin Deraedt (XPE Pharma & Science on behalf of GSK).
